# Do older adults still choose comfortable cities? The quality of life and its affect on Indonesia’s older adult population

**DOI:** 10.3389/fpubh.2025.1480485

**Published:** 2025-08-21

**Authors:** Sri Subanti, Arif Rahman Hakim, Mas Rahmah, Asri Laksmi Riani, Hasih Pratiwi, Juansih Juansih, Wahyu A. P. M. Wibawa, Sendy A. M. Uktutias

**Affiliations:** ^1^Universitas Sebelas Maret, Surakarta, Indonesia; ^2^Universitas Airlangga, Surabaya, Indonesia

**Keywords:** older adults, quality of life, cities, comfortable, Indonesia

## Abstract

**Introduction:**

Maintaining the quality of life for the aging urban population is becoming more and more crucial since this is becoming a global phenomenon. Cities need to be comfortable and accommodate facilities according to the needs of older adults to support the aging of the urban older adults while improving their quality of life. This paper aims to determine the value of a city’s quality of life in relation to its urban facilities and services, as well as to examine the relationship between quality of life and the aging population in Indonesian cities.

**Methods:**

This study measures the quality of life using a hedonic model approach, which determines the price of each component of city comfort, both as facilities and services. We also employed an empirical model to investigate the relationship between quality of life and the aging population in Indonesian cities.

**Results:**

Our research shows that the majority of Javan cities have high quality of life values because older residents have lived there for a long time, they can access facilities and services that meet their needs as they age.

**Discussion:**

The quality of life plays an important role in the number of urban older adults, with health facilities, older adults-friendly transportation, security guarantees, and communication accessibility having a significant effect on the increase in urban older adults in Indonesia. The city governments should provide urban facilities that understand the characteristics and adjust to the needs of older adults.

## Introduction

The global older adults population will continue to grow, as life expectancy and birth rates increase. Recent estimates project that by 2050, older adults will account for 22 percent of the world’s population, or around 2.1 billion people will be aged 60 and over. For example, East and Southeast Asia is projected to have 572.5 million older adults aged 65 + by 2050. Urban areas are experiencing an increase in older adults population, with more than 60 percent of the global older adults living in cities, driven by the trend of urbanization (1). In Indonesia, conditions are not much different with the trend of aging accelerating. Older adults population is projected to grow from 10.1% in 2020 to 18.0% in 2040, with a higher proportion of women. By 2050, the United Nations estimates that 25% of Indonesia’s population, around 74 million people, will be older adults, a significant increase from 6.86% in 2022 (2). In line with this projection, older adults will reach 20% of the population by 2040, up from 7.6% in 2010. Indonesia’s total population is expected to reach 320.7 million by 2050, a 14% increase from 281.2 million in 2023, with older adults segment growing disproportionately (3).

In the context of aging urban populations as a result of demographic shifts are being guided by a declining birth rate, shrinking households, rising healthcare costs, and fragmentation among traditional family structures (4). A city, defined as a densely populated area surpassing non-urban regions in population, size, or significance (5), cities face a growing older adults population then urban design must enhance older adults quality of life by supporting aging in place (6). Older adults citizens require accessible facilities to maintain independence and perform daily tasks (7). As urban centers advance, many older adults still have experienced neglect from inadequate access to healthcare services, increasing insecurity due to rising crime rates, communication infrastructure which restricts their engagement with digital platforms, and reliable public transportation (8, 9). This highlights the critical need for policymakers to design age-friendly cities that ensure comfort and mobility for older adults (10).

Research highlights the importance of features for older adults such as healthcare services, transportation, communication, safety, and low-crime environments (11–14). Health facilities are very important for older people, especially those with chronic conditions or functional limitations, while accessible health services improve disease prevention and overall health (15) also it can support physical health and well-being in improving their quality of life (16, 17). Transportation should be easily accessible and be a foundation for older people to increase their independence, facilitate access to medical and social services, and foster a sense of autonomy that contributes to their psychological health (18–20). Good communication infrastructure encourages involvement in social activities while reducing the potential for isolation and has the potential to improve emotional health, which is important in improving a more satisfying quality of life in the future (21). An environment that provides a sense of security can provide confidence to actively participate in daily life, thereby increasing life satisfaction (19, 22). Similarly, low crime provides a sense of security in ensuring that older adults can remain engaged in community activities while strengthening social connectedness and overall quality of life (23, 24). Collectively, these interrelated factors can create an ecosystem that supports and empowers older adults to live healthier, more engaged, and fulfilling lives.

As the aging population grows, developing comfortable and inclusive cities to support older adults must become a key public policy priority. There are not many studies that identify this factor in the context of older adults, especially in developing countries, including Indonesia. This research wants to contribute by calculating the city comfort aspects in the context of quality of life from the perspective of older adults, by accommodating specific groups of city facilities needed by them. The findings can highlight the value of older adults as a resource and urge urban development policies must to create more age-friendly cities. The government need address demographic aging by fostering cities that optimize their potential.

## Methodology

### The framework for measuring quality of life

This study measures the quality of life by referring to the basic framework built by Rosen (25) and Roback (26), analyzing the differences in compensation and quality of life based on the assumption that consumers or workers have the same preferences and companies are subject to technological similarities in facing local convenience bundles between cities. Spatial equilibrium conditions allow for the absence of incentives to move, where compensation for individuals or households living in cities with high quality of life will face a tendency for low wages and high housing rents. Conversely, those who get a low quality of life encounter a tendency for high wages and low rents for housing. The form of compensation that must be paid and compensation that must be received is the value of local convenience called the quality of city life.

The model comprises two economic agents: households and firms. Each household consists of one worker who is employed by a local firm and earns a wage (w). Households aim to maximize their utility through consumption, subject to a budget constraint involving commodity goods (G) which is assumed to be priced at 1, and housing (H), which provides comfort (A) at a chosen location. This maximization problem can be expressed mathematically as follows.


(1) maxU(G,H;A)s.t.w+I=G+Hr

The variables w and I represent wages and non-labor income, respectively. Through optimization, the optimal levels of consumption (X*) and housing (H*) are derived. By substituting these into the initial utility function, the indirect utility function (V) is obtained. The market equilibrium condition for workers as follows.


(2) V(w,r;A)=k

The equation shows that increasing comfort will increase utility if A is the comfort consumed by the consumer $\left(V_{A}>0\right)$ . Utility decreases if A is an inconvenience for the consumer $\left(V_{A}>0\right)$ and will not affect if A is not categorized as a comfort factor $\left(V_{A}=0\right)$ . Wage and rent adjustments need to be adjusted to ensure equal utility in all locations, so that there is no incentive to move to another city. In other words, if wages increase, it will be followed by an increase in utility for those who work in the city, so that to keep them living or not migrating to another city, it will be followed by an increase in housing rent. Likewise, an increase in housing rent will drive utility down, so that they do not move, it will be followed by an increase in wages. Thus, the utility of those who live in one city and another city will be the same and migration between cities does not occur.

On the firm side, the production function is applied by assuming constant returns to scale, where L is the land used for production and N is the number of workers; the production function for goods (G) is


(3) G=f(L,;N,;A)

Through optimization, firms minimize production costs subject to production function constraints, yielding optimal solutions for labor (N*) and other inputs (L*). The equilibrium condition for the company is achieved when the production cost per unit is the same as the product price, expressed as follows.


(4) C(w,r;A)=k

The equation represents the equilibrium condition, which is intended so that there is no incentive for companies to relocate production activities to other cities. The company’s cost function will increase along with the increase in production factors, namely $C_{w},C_{r}>0$ with $C_{w}=N$ , $C_{r}=L$ . The decrease in production costs if A is a convenience for the company/producer $\left(C_{A}<0\right)$ , the increase in production costs will be borne by the company if A is an inconvenience $\left(C_{A}>0\right)$ , and there is no effect on production costs when A is not a convenience for the company $\left(C_{A}=0\right)$ . Wages and rents will be adjusted so that the production costs borne by the company are the same between cities. If workers’ wages increase, it will be followed by an increase in the firm’s production costs, so that to keep them from relocating their factories to other cities, it will be followed by a decrease in housing rents. Likewise, an increase in housing rents will drive up production costs, so that no relocation will be followed by a decrease in wages. Thus, production costs in one city and another city will be the same and firm relocation between cities will not occur. Next, the effect of convenience on the balance of wages and land rents is obtained as follows.


(5) $$V_{w}\mathrm{dw}+V_{r}\mathrm{dr}+V_{A}\mathrm{dA}=0\to V_{w}\frac{\mathrm{dw}}{\mathrm{dA}}+V_{r}\frac{\mathrm{dr}}{\mathrm{dA}}+V_{A}=0$$

(6) $$C_{w}\mathrm{dw}+C_{r}\mathrm{dr}+C_{A}\mathrm{dA}=0\to C_{w}\frac{\mathrm{dw}}{\mathrm{dA}}+C_{r}\frac{\mathrm{dr}}{\mathrm{dA}}+C_{A}=0$$

Based on the above two equations, a matrix can be formed as $\left[\begin{array}{cc} V_{w} & V_{r}\\ C_{w} & C_{r} \end{array}][\begin{array}{c} \frac{\mathrm{dw}}{\mathrm{dA}}\\ \frac{\mathrm{dr}}{\mathrm{dA}} \end{array}]=[\begin{array}{c} -V_{A}\\ -C_{A} \end{array}\right]$ , then solving this yields $\frac{\mathrm{dw}}{\mathrm{dA}}=\frac{-V_{A}C_{r}+V_{r}C_{A}}{V_{w}C_{r}-V_{r}C_{w}}$ and $\frac{\mathrm{dr}}{\mathrm{dA}}=\frac{-V_{w}C_{A}+V_{A}C_{w}}{V_{w}C_{r}-V_{r}C_{w}}$ . By reorganizing into $\left(\frac{\mathrm{dw}/\mathrm{dA}}{\mathrm{dr}/\mathrm{dA}}\right)$ , an equation is derived that shows how relative changes in wages and housing rents respond to variations in comfort (A), depending on the relative importance of housing versus labor in production costs. When labor is more significant than housing in production, housing rents tend to be more sensitive to changes in comfort than wages. It’s applied to analyze the location decision against the measurement of household or individual willingness to pay for convenience. In the Rosen & Roback model, the willingness to pay solution $\left(V_{A}/V_{w}\right)$ is shown by


(7) $$\frac{V_{A}}{V_{w}}=y^{*}\frac{\mathrm{dr}}{\mathrm{dA}}-\frac{\mathrm{dw}}{\mathrm{dA}}=Z_{j}$$

In the literature, it is assumed that each household consumes exactly one housing unit, denoted as $y^{*}$ , representing the equilibrium housing consumption. This equation quantifies the monetary amount a household must pay or receive for the comfort provided by a city. The quality of life (QoL) index is formulated as follows.


(8) $$\mathrm{QoL}=\sum Z_{j}a_{j}$$

Here, $a_{j}$ represents the set of comfort variables present in city *j*. The quality-of-life index, ${\mathrm{QoL}}_{j}$ , is defined as the sum of these comforts, reflecting the total estimated compensation that households for comfort in a city through the housing market and labor market.

### The empirical model

In this study, 98 Indonesian cities’ older adults quality of life indices were computed in Indonesia (see Appendix 1 for map of cities in Indonesia). The Quality of Life takes into account the facilities that older people require in cities, and it covers the following aspects: economy, health facilities, crime, security, transportation, pollution, open public spaces, and communication. The information needed includes information on wages, rent, and the characteristics of older adults, the condition of houses, and city facilities. The Central Statistics Agency provided the PODES (Village Potential) statistics and the SUSENAS (National Socioeconomic Survey) data for other information regarding city amenities. This study uses both data published simultaneously in 2021, which is the latest publication published by the Indonesian Central Statistics Agency.

The obtained index values will provide us with information on which cities elders find more appealing, comfortable, and desirable than others. We employed an empirical model that was adjusted for this study in order to assess the relationship between the older population as a proxy for the percentage of older adults people and the city facilities or services represented by the Quality of Life Index.


(9) $$\text{older adults}=\gamma_{1}+\gamma_{2}\;\mathrm{QoL}+\gamma_{3}\;\text{Control}+e\;$$

The dependent variable (older adults) states the proportion of older adults people as the dependent variable, the information for which was obtained from the Central Statistics Agency. Quality of life and control variables represent the independent variables. From the perspective of older adults, the following some component from city comfort can determine a city’s quality of life ( QoL ): health, communication, transport, crime, and security. Additionally, the density of older adult citizens per square kilometer is the control variable that is employed. Both the independent and dependent variables that are included in the model share the same time period.

## Results and discussion

Table 1 displays the results of the calculation of the city quality of life index by region. Cities in Java and Bali have the highest average values. These findings show that the quality of life for older adults in cities in Java and Bali is better than in cities in other regions. Conversely, cities located in the provinces of Papua, West Nusa Tenggara, East Nusa Tenggara, Maluku, North Maluku, and Papua are part of other regional groups. Cities in this area offer a range of relatively similar city comforts to elders who prefer to live there. The Java & Bali region has the largest variation in quality of life between regions. The highest variation in quality of life between regions is found in Java & Bali, where the comfort of cities is supportive of the development of comfort cities for older adults by paying attention to their needs, particularly in terms of facilitating health services, allowing them to engage in independent economic activity, providing open public spaces, and ensuring that easily accessible transportation is affordable (27–29). In addition, these cities must give older adults a sense of security so they may go about their everyday lives and be backed by enough security guards to reduce the possibility that they will become victims of crimes (10).

**Table 1:** Quality of life based on region.

Region	Number of cities	Avg.	Std. Dev	Min		Max	
				Value	City	Value	City
Sumatra	34	18.87	6.90	6.71	Gunungsitoli	33.10	Palembang
Java & Bali	35	40.15	10.94	20.31	Serang	60.17	Bandung
Kalimantan	9	24.93	7.99	10.64	Singkawang	36.41	Bontang
Sulawesi	11	18.76	6.58	9.01	Palopo	30.81	Makassar
Others	9	18.26	4.31	11.59	Tual	24.78	Jayapura
Indonesia	98	26.96	13.09	6.71	Gunungsitoli	60.17	Bandung

A comfortable city should be able to accommodate the needs of older adults residents, as this can improve their quality of life and naturally provide them comfort and satisfaction (30). According to the results of the Quality of Life calculations, the cities on Java Island tend to be more comfortable for people to live in and age in place since these cities can accommodate their activities with all the limitations that come with getting older. From the perspective of the perspective of older adults, the city should be able to recognize their needs and adapt its layout to provide the facilities they require (31, 32). Complete health facilities, easy access to communication, a sense of security for them to carry out their daily activities, and easily accessible modes of transportation are some of the facilities that can make older adults people feel comfortable living in the city. and interacting with others (6, 14, 33, 34). Older adults can live well in several non-Javanese cities, as seen by the high quality of life values according to area, including Bandar Lampung, Balikpapan, and Makassar. These are the provincial capitals and have attracted a large number of migrants, allowing for rapid development of cities and the provision of a wide range of additional facilities, including hotels, schools, retail stores, health facilities, and places to entertain (35).

However, developing a city that becomes “home” for all of society, especially older adults, frequently requires high development costs and the determination of regional leaders to make it through (5). This situation frequently arises when the majority of local governments still depend extensively on the national government, particularly when it comes to building mass transportation that is reasonably priced, health facilities, and other centers of economic activity like marketplaces. Since the government cannot afford to provide these facilities, the private sector must play a part in developing infrastructure that can make the city comfortable while improving its quality of life (PwC (36)).

**Figure 1:**
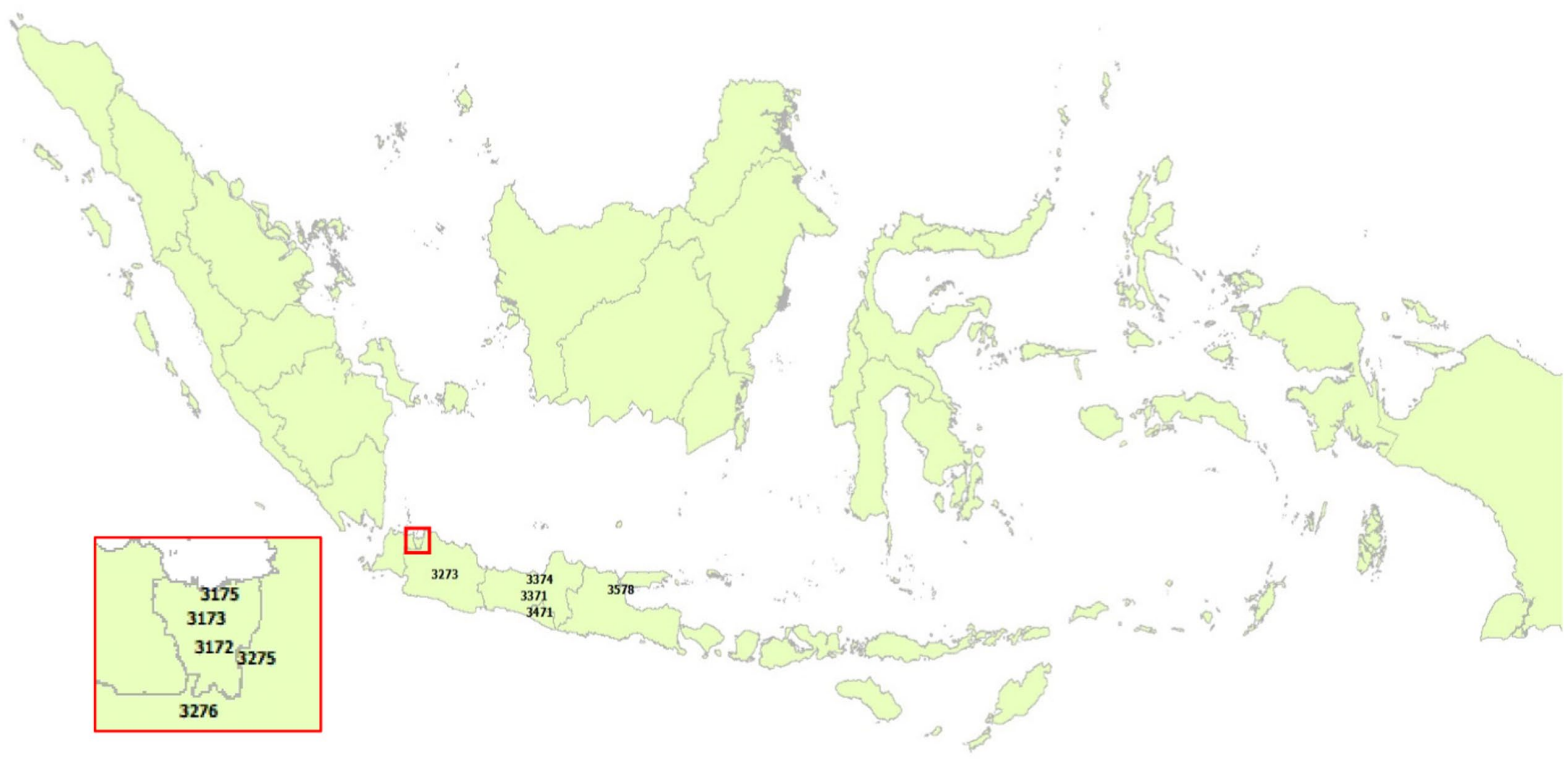
Cities in the top 10.

**Figure 2:**
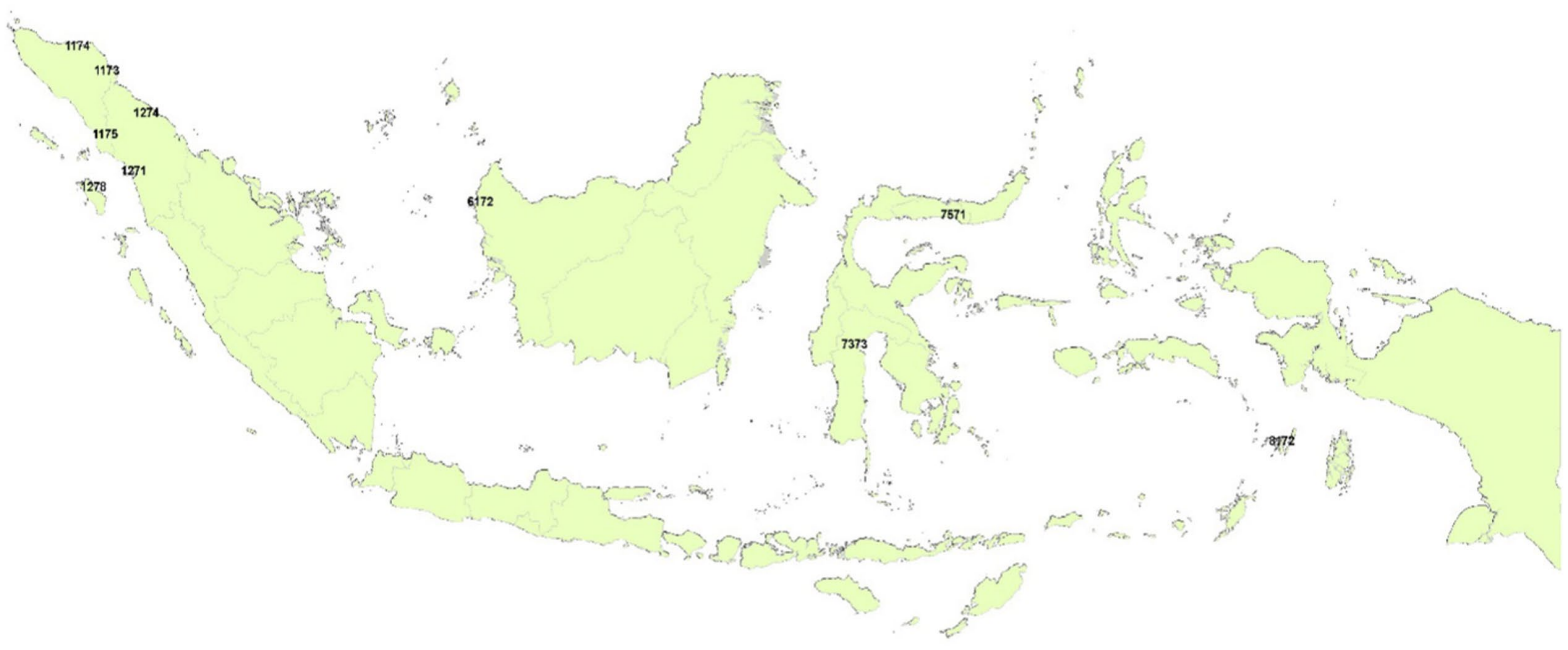
Cities in the bottom 10.

Table 2 presents the results of calculating the QoL Index for cities for older adults in Indonesia. We only present the 10 highest and 10 lowest cities, while the complete calculation results can be seen in Appendix 2. We also present the cities for the 10 highest and 10 lowest categories on the map (Figures 1, 2). Cities on Java Island still dominate this category with cities in DKI Jakarta Province contributing three cities, namely East Jakarta, North Jakarta, and Central Jakarta; then West Java Province is Bandung and Bekasi. The cities in Central Java are Semarang and Magelang, while the Special Region of Yogyakarta Province is Yogyakarta City. Surabaya represents a city from East Java Province in this category. The city of Bandung earned the highest value where this value reflects the price that must be paid by households or older adults individuals implicitly, which is 60.17 million rupiah, through the housing market and labor market in order to be able to utilize the facilities that make living in the city comfortable.

**Table 2:** The quality of life based on 10 highest city and 10 lowest city.

10 highest city			10 lowest city		
Code	City	Value	Code	City	Value
3273	Bandung	60.17	1274	Tebing Tinggi	12.24
3172	East Jakarta	59.20	8172	Tual	11.59
3275	Bekasi	57.25	1271	Sibolga	11.29
3578	Surabaya	55.90	6172	Singkawang	10.64
3175	North Jakarta	53.40	7571	Gorontalo	9.57
3471	Yogyakarta	52.17	1173	Langsa	9.27
3371	Magelang	52.08	7373	Palopo	9.01
3374	Semarang	51.65	1175	Subulussalam	8.44
3276	Depok	48.32	1174	Lhokseumawe	8.26
3173	Central Jakarta	46.86	1278	Gunungsitoli	6.71

The findings also show that the cities included in the 10th highest category are dominated by cities that are capitals, both provincial capitals and national capitals, such as the cities of the DKI Jakarta Province, Bandung, Semarang, Yogyakarta, and Surabaya. It demonstrates that Indonesia’s development is still concentrated on a few main cities, particularly the capitals that serve as centers for commerce, industry, and services, as well as excellent providers of financial networking services. Consequently, these cities become popular places for individuals to move to and live permanently (37). With complete health facilities, convenient public transportation, plenty of open public spaces for their activities, complete economic activity centers, and ease of correspondence with family members, residents of cities have many advantages that make them less likely to want to move when they are older (38, 39).

Cities that can support the state capital, like Bekasi and Depok, have the ability to provide support for older adults because their residents have lived there for a long time. The city government also contributes to the improvement of the citizens’ quality of life by offering public services and facilities that can accommodate the residents (40). Next, this category includes Magelang, which is a representation of a small city in the province of Central Java. Older adults population in these cities consists primarily of retired state and private public servants who chose the region for its peace, quiet, minimal pollution, strong social links among residents, and generally reasonable cost of living (41–43). The characteristics of cities often became retirement destinations for those who previously lived in big cities or capitals, giving the city the label of “Retirement City” (44).

There are two cities that are the result of regional expansion, namely Gunung Sitoli and Subulussalam. The availability of open public spaces, the incompleteness of economic activity centers, the need for improved communication access, and the lack of community mobility supported by public transportation are just a few of the facilities that these cities are still unable to provide in order to fully serve the needs of older adults. These cities are still unable to build complete city facilities to accommodate the interests of older adults, such as the availability of open public spaces, the incompleteness of economic activity centers, the need for improved communication access, and the lack of older adults mobility supported by public transportation. Being the capital of a province that is part of this group, Palu must give older adults a sense of security when they go about their daily activities so they will not have to worry about possible crimes that could happen to them. In general, cities in this group need to accommodate the facilities needed by older adults in their city development, such as health, security, transportation, economic activity, and the availability of public space (11, 14, 45).

Based on the empirical estimation results presented in Table 3, we found that several components of quality of life significantly influence older adults population in Indonesian cities. The quality of life component for health has a positive effect and can increase the number of the city’s older adults population living and aging in a city. Due to the natural aging process, older people have higher health demands than younger people. As a result of multiple complicated health disorders emerging simultaneously, they often experience a decrease in multiple illnesses at once, which is commonly referred to as geriatric syndromes (46). Older adults require more comprehensive health facilities that can suit their needs because their physical and mental health tends to worsen with age (47). The older population frequently needs intensive care, which is frequently unavailable at home due to their high average length of hospital stays. In addition, this population group stays longer on average than other population groups. Consequently, the overall need for health services will rise as the number of older adults people increases (16). The city governments must persist in offering public health facilities and services that prioritize the needs of older adults and make use of their abilities and well-being (10). Older adults need support so that they can enjoy an active and independent life so that their quality of life increases when they decide to live and grow old in the city (48).

**Table 3:** Regression output.

DEP: older adultsperc.	Model 1		Model 2		Model 3	
INDEP	Coef.	Sign.	Coef.	Sign.	Coef.	Sign.
health	0.307	***	0.304	***	0.310	***
communication	0.196	***	0.204	***	0.162	***
transport	0.020		0.027		0.005	
crime	−0.038	**	−0.035	*	−0.039	*
security	0.014	*	0.014	*	0.020	***
control	0.011		0.004		0.008	
constant	−1.258	***	−1.304	***	−1.023	
N	98		91		83	
F	54.120		49.500		53.760	
Prob F	0.000		0.000		0.000	
R-Sq	0.902		0.901		0.911	

***sign. α = 1%; **sign. α = 5%; *sign. α = 10%.

Security is a component of quality of life that older adults are concerned about because it positively affects older adults population, based on our empirical results. Older adults make safety an important factor for them to be able to age actively. The urban environment in which they age must be safe, inclusive for older adults, and have easy access that accommodates a variety of needs (49, 50). Security can promote older people’s mobility since a safe environment will allow them to engage in optional activities that are dependent upon their requirements and how they use the space (51). Older adults people who are comfortable with nature, urban areas, and parks are more likely to select outside activities. In contrast, older adults will choose activities with lots of people around them when they feel otherwise (52).

Older adults will be less interested in residing in a city if there is a high crime rate. Older adults are aware of the elements of crime that could lurk and affect them personally. Older adults people are frequently victims of crimes like aggression, assault, and robbery (24). Older adults who experience extreme anxiety often cut back on their walking to lessen their chance of becoming victims of crime (62). When older adults individuals perceive criminality in their surroundings, they often restrict their mobility or choose different routes if they choose to continue participating in outdoor activities (53). This is because older adults are vulnerable to street crimes such as bag snatching and robbery, so this becomes something frightening for them (54). The aging process has become a natural part of life, which lowers their physical strength and dexterity and makes their body less effective at resisting injury. As a result, they are more vulnerable than young people. Older adults people are susceptible to severe consequences from even slight injuries, including considerable psychological effects and possibly lifelong harm (55).

The better a city’s communication infrastructure provided by local government will make older adults settle and attract other older adults people to choose to live in that area, as a result, the number of older adults tends to increase (56). Older adults will find it easier to communicate information about their condition using voice messages, photos, videos, particular application platforms, and other forms connected to their everyday contacts if communication facilities are improved (21). Encouraging communication will help them stay in touch with their families, their current health, and their daily needs. The more complete communication technology can make it easier for older adults to overcome social and spatial obstacles because they can expand the reach of interaction in various forms of activities anytime and anywhere. The usage of computer and internet media, which require good signals, can help older people who are socially and familiarly lonely by providing them with good communication capabilities (57). Older adults require social support in order to engage with others because sickness and diminished physical capacity are intimately associated with aging. The support of good communication facilities will allow them to make video calls or teleconferences with their families and supporting communities (58, 59). Furthermore, more complete and better communication facilities have the potential to help older adults adapt to new environments such as nursing homes (60). Older adults can also release feelings of control and avoid losing self-esteem because they can still carry out their daily routines and engage in the activities they usually do (61).

## Conclusion

Cities should be able to accommodate the various needs of their residents, which often change based on the demographics of the region. In the context of older adults, they often choose to live and aging in cities that can accommodate their needs for services and facilities because they believe these communities can enhance their quality of life. Studies that connect various aspects of city quality of life with older adults population are still limited, and they do not accurately reflect situations in developing countries. This study aims to calculate the value of the quality of life of cities in relation to services and facilities that cater to the requirements of older adult citizens, including public health, public spaces, security, pollution, communication, and transportation. We also examine the relationship between quality of life and older adults population by providing evidence from Indonesia.

Our study results found that the majority of cities with facilities and infrastructure that support the daily activities of older adults are found on Java Island. Cities such as Bandung, Surabaya, East Jakarta, Bekasi, Depok, Yogyakarta, and Semarang are categorized as cities with high quality of life values, followed by the increasing number of older adults people choosing to live there. These cities have become destinations for older adults because they have lived there for a long time to work and decided to grow old there. These cities are also able to provide support for older adults, especially in improving the quality of life by providing public services and facilities that can accommodate their needs. Cities such as Magelang, Cirebon, Salatiga, Tasikmalaya, Batu, Denpasar, and Balikpapan have the potential to become destinations for older adults to grow old in, considering that these cities are included in the category of cities with a high quality of life.

Cities with a higher quality of life will make older adults choose to live and age in a city, thereby increasing the number of older adults in that city. Older adults who decide to live in cities with a high quality of life do so because it can offer them a comfortable life and cater to their needs, which are directly tied to their restrictions. Older adults people may feel less of a desire to move because cities already provide high-quality healthcare facilities that meet their needs, accessible public transportation that takes into account their unique needs, a sense of security for their everyday activities, public areas where they can socialize and meet new people, well-developed economic hubs, and the ease of communicating to convey news to family and colleagues.

Our study provides suggestions for city governments to create an ideal city for older adults by understanding their characteristics so that city planning can adapt to the needs of older adults. City facilities and services that accommodate the needs of older adults can make them happier and more comfortable to live in while improving the quality of life in their old age. This study has limitations in the availability of data, especially for facilities or services that are closely related to older adults, such as nursing homes, older adults care centers, and libraries, where this information is not available in the data used in this study. Future studies can add city facilities and services related to older adults to improve the quality of life in the city.

## Data Availability

The raw data supporting the conclusions of this article will be made available by the authors, without undue reservation.

## Supplementary material

SUPPLEMENTARY APPENDIX 1 The quality of life in Indonesian cities.
