# Supplementary material for: Do older adults still choose comfortable cities? The quality of life and its affect on Indonesia’s older adult population
Source: Front Public Health. 2025 Aug 21;13:1480485. doi: 10.3389/fpubh.2025.1480485 (PMC12409721; doi:10.3389/fpubh.2025.1480485)
Supplement: SUPPLEMENTARY APPENDIX 1 — The quality of life in Indonesian cities. [file Table_1.pdf]

APPENDIX 1. THE QUALITY OF LIFE BASED ON INDONESIAN CITIES

no	idcity	cityname	idprovince	provincename	QoL
1	1171	BANDA ACEH	11	SPECIAL REGION OF ACEH	16.04
2	1172	SABANG	11	SPECIAL REGION OF ACEH	22.58
3	1173	LANGSA	11	SPECIAL REGION OF ACEH	9.27
4	1174	LHOKSEUMAWE	11	SPECIAL REGION OF ACEH	8.26
5	1175	SUBULUSSALAM	11	SPECIAL REGION OF ACEH	8.44
6	1271	SIBOLGA	12	NORTH SUMATERA	11.29
7	1272	TANJUNG BALAI	12	NORTH SUMATERA	15.68
8	1273	PEMATANG SIANTAR	12	NORTH SUMATERA	12.52
9	1274	TEBING TINGGI	12	NORTH SUMATERA	12.24
10	1275	MEDAN	12	NORTH SUMATERA	31.58
11	1276	BINJAI	12	NORTH SUMATERA	16.78
12	1277	PADANGSIDIMPUAN	12	NORTH SUMATERA	15.42
13	1278	GUNUNGSITOLI	12	NORTH SUMATERA	6.71
14	1371	PADANG	13	WEST SUMATERA	27.70
15	1372	SOLOK	13	WEST SUMATERA	28.65
16	1373	SAWAH LUNTO	13	WEST SUMATERA	15.83
17	1374	PADANG PANJANG	13	WEST SUMATERA	20.69
18	1375	BUKITTINGGI	13	WEST SUMATERA	12.82
19	1376	PAYAKUMBUH	13	WEST SUMATERA	15.50
20	1377	PARIAMAN	13	WEST SUMATERA	22.40
21	1471	PEKANBARU	14	RIAU	26.86
22	1473	D U M A I	14	RIAU	16.51
23	1571	JAMBI	15	JAMBI	30.61
24	1572	SUNGAI PENUH	15	JAMBI	15.37
25	1671	PALEMBANG	16	SOUTH SUMATERA	33.10
26	1672	PRABUMULIH	16	SOUTH SUMATERA	16.61
27	1673	PAGAR ALAM	16	SOUTH SUMATERA	19.66
28	1674	LUBUKLINGGAU	16	SOUTH SUMATERA	20.08
29	1771	BENGKULU	17	BENGKULU	24.55
30	1871	BANDAR LAMPUNG	18	LAMPUNG	22.69
31	1872	METRO	18	LAMPUNG	20.50
32	1971	PANGKAL PINANG	19	BANGKA BELITUNG ISLANDS	15.67
33	2171	B A T A M	21	RIAU ISLANDS	26.15
34	2172	TANJUNG PINANG	21	RIAU ISLANDS	22.91
35	3171	SOUTH JAKARTA	31	SPECIAL CAPITAL REGION OF JAKARTA	42.17
36	3172	EAST JAKARTA	31	SPECIAL CAPITAL REGION OF JAKARTA	59.20
37	3173	CENTRAL JAKARTA	31	SPECIAL CAPITAL REGION OF JAKARTA	46.86
38	3174	WEST JAKARTA	31	SPECIAL CAPITAL REGION OF JAKARTA	42.50
39	3175	NORTH JAKARTA	31	SPECIAL CAPITAL REGION OF JAKARTA	53.40
40	3271	BOGOR	32	WEST JAVA	36.95
41	3272	SUKABUMI	32	WEST JAVA	37.39
42	3273	BANDUNG	32	WEST JAVA	60.17

no	idcity	cityname	idprovince	provincename	QoL
43	3274	CIREBON	32	WEST JAVA	42.76
44	3275	BEKASI	32	WEST JAVA	57.25
45	3276	DEPOK	32	WEST JAVA	48.32
46	3277	CIMAHI	32	WEST JAVA	34.72
47	3278	TASIKMALAYA	32	WEST JAVA	40.45
48	3279	BANJAR	32	WEST JAVA	40.37
49	3371	MAGELANG	33	CENTRAL JAVA	52.08
50	3372	SURAKARTA	33	CENTRAL JAVA	32.22
51	3373	SALATIGA	33	CENTRAL JAVA	40.71
52	3374	SEMARANG	33	CENTRAL JAVA	51.65
53	3375	PEKALONGAN	33	CENTRAL JAVA	24.05
54	3376	TEGAL	33	CENTRAL JAVA	22.95
55	3471	YOGYAKARTA	34	SPECIAL REGION OF YOGYAKARTA	52.17
56	3571	KEDIRI	35	EAST JAVA	34.02
57	3572	BLITAR	35	EAST JAVA	28.26
58	3573	MALANG	35	EAST JAVA	46.62
59	3574	PROBOLINGGO	35	EAST JAVA	20.71
60	3575	PASURUAN	35	EAST JAVA	25.48
61	3576	MOJOKERTO	35	EAST JAVA	27.28
62	3577	MADIUN	35	EAST JAVA	35.25
63	3578	SURABAYA	35	EAST JAVA	55.90
64	3579	BATU	35	EAST JAVA	41.44
65	3671	TANGERANG	36	BANTEN	37.55
66	3672	CILEGON	36	BANTEN	31.43
67	3673	SERANG	36	BANTEN	20.31
68	3674	SOUTH TANGERANG	36	BANTEN	43.70
69	5171	DENPASAR	51	BALI	39.08
70	5271	MATARAM	52	WEST NUSA TENGGARA	19.70
71	5272	BIMA	52	WEST NUSA TENGGARA	22.37
72	5371	KUPANG	53	EAST NUSA TENGGARA	18.46
73	6171	PONTIANAK	61	WEST KALIMANTAN	24.80
74	6172	SINGKAWANG	61	WEST KALIMANTAN	10.64
75	6271	PALANGKA RAYA	62	CENTRAL KALIMANTAN	16.57
76	6371	BANJARMASIN	63	SOUTH KALIMANTAN	20.77
77	6372	BANJAR BARU	63	SOUTH KALIMANTAN	28.61
78	6471	BALIKPAPAN	64	EAST KALIMANTAN	35.43
79	6472	SAMARINDA	64	EAST KALIMANTAN	29.20
80	6474	BONTANG	64	EAST KALIMANTAN	36.41
81	6571	TARAKAN	65	NORTH KALIMANTAN	21.91
82	7171	MANADO	71	NORTH SULAWESI	24.35
83	7172	BITUNG	71	NORTH SULAWESI	19.06
84	7173	TOMOHON	71	NORTH SULAWESI	20.93
85	7174	KOTAMOBAGU	71	NORTH SULAWESI	18.00
86	7271	PALU	72	CENTRAL SULAWESI	13.91
87	7371	MAKASSAR	73	SOUTH SULAWESI	30.81

no	idcity	cityname	idprovince	provincename	QoL
88	7372	PAREPARE	73	SOUTH SULAWESI	27.48
89	7373	PALOPO	73	SOUTH SULAWESI	9.01
90	7471	KENDARI	74	SOUTHEAST SULAWESI	14.77
91	7472	BAUBAU	74	SOUTHEAST SULAWESI	18.46
92	7571	GORONTALO	75	GORONTALO	9.57
93	8171	AMBON	81	MALUKU	22.22
94	8172	TUAL	81	MALUKU	11.59
95	8271	TERNATE	82	NORTH MALUKU	12.31
96	8272	TIDORE ISLANDS	82	NORTH MALUKU	17.81
97	9171	SORONG	91	WEST PAPUA	15.08
98	9471	JAYAPURA	94	PAPUA	24.78
